# Artificial Intelligence in Postharvest Food Safety Control of Animal-Source Foods: Evidence Thresholds, Validation, and Regulatory Applicability

**DOI:** 10.3390/vetsci13060574

**Published:** 2026-06-11

**Authors:** András Bittsánszky, Vilmos Bilicki, Gergő Sudár, Miklós Süth, Szilvia Kusza, András J. Tóth

**Affiliations:** 1 Department of Food Hygiene, Institute of Food Chain Science, University of Veterinary Medicine Budapest, István u. 2., H-1078 Budapest, Hungary; bittsanszky.andras@univet.hu (A.B.); toth.andras.jozsef@univet.hu (A.J.T.); 2 Department of Software Engineering, Institute of Informatics, University of Szeged, H-6720 Szeged, Hungary; bilickiv@inf.u-szeged.hu; 3 CERES Holding Ltd., 7030 Paks, Hungary; gergo.sudar@ceresholding.eu; 4 Centre for Agricultural Genomics and Biotechnology, Faculty of Agricultural and Food Sciences and Environmental Management, University of Debrecen, Egyetem tér 1, H-4032 Debrecen, Hungary; kusza@agr.unideb.hu

**Keywords:** artificial intelligence, animal-source foods, postharvest food safety, narrative review, external validation, HACCP, traceability, regulatory applicability

## Abstract

**Background**: Artificial intelligence (AI) is increasingly being proposed for postharvest food-safety control of animal-source foods, but its practical value depends on whether models can support real decisions rather than only report high accuracy. **Methods**: This narrative review used a structured literature mapping of peer-reviewed work, mainly from 2020 to 2025, identified through database searches and citation tracking using combined terms for artificial intelligence, machine learning, animal-source foods, postharvest food safety, slaughterhouse inspection, cold-chain monitoring, traceability, authenticity, HACCP, validation, and regulatory applicability. **Results**: The most implementation-proximate applications are computer vision prescreening in slaughterhouses and processing plants, sensor- and IoT-based cold-chain surveillance, freshness and adulteration screening, and digital traceability systems. Across these areas, stronger evidence is associated with clearly defined control points, transparent reference methods, external or temporal validation, auditable data flows, and documented human oversight. The main weaknesses are single-site datasets, retrospective designs, incomplete reporting of reference methods, limited workflow testing, and insufficient attention to false alerts, fallback procedures, and governance. **Conclusions**: AI should be viewed as targeted decision support, not as a replacement for established food-safety control. Future studies should prioritize prospective, multi-site, workflow-embedded validation and show how alerts lead to documented corrective or verification actions.

## Simple Summary

Artificial intelligence is often presented as a major opportunity for safer food production, but many published systems are still far from routine use. This review focuses on animal-source foods after harvest or slaughter, including meat, milk, eggs, and seafood, and asks where AI can realistically strengthen food-safety control. The strongest current opportunities are not broad autonomous systems, but targeted tools: camera-based prescreening in slaughterhouses and processing plants, sensors that detect cold-chain problems, digital systems that improve traceability and recalls, and HACCP tools that document alerts and corrective actions. The review also shows why high model accuracy is not enough. A useful system must be tested beyond one dataset or one plant, compared with a credible reference method, connected to a clear control decision, and supported by human oversight and an audit trail. The main message is that AI can improve speed, consistency, and documentation, but only when it is validated in realistic conditions and built into existing food-safety workflows.

## 1. Introduction

Foodborne diseases continue to impose a substantial public health and economic burden worldwide, and that burden is particularly high where regulatory capacity, analytical infrastructure, or documented traceability are weak [1,2]. Animal-source foods require special attention because zoonotic reservoirs, high water activity, multiple processing steps, and strong dependence on the cold chain jointly increase microbiological and certain chemical risks [3,4]. In meat products, dairy products, egg products, and fishery products, delayed detection or poor lot identification can rapidly escalate into geographically widespread incidents, recalls, or trade disruption.

Interest in AI and digital food-safety systems is therefore understandable. The literature describes the rapid spread of real-time sensor data, machine learning, computer vision, digital traceability, and decision-support platforms, and many publications argue that these tools can shift food-safety systems from reactive inspection toward proactive, risk-based control [5,6,7,8,9,10]. At the same time, rapid technological expansion can obscure a more important question: whether a given model or tool is actually suitable for official control, inter-plant comparison, HACCP verification, or real-time operational decision support.

For postharvest food-safety control, the key question is therefore not “what technologies exist”, but which applications are supported by evidence that is paired with human food-safety benefit, validated performance, auditable operation, and a realistic path to implementation. Accordingly, this review shifts attention away from the broader digitalization narrative and toward postharvest control settings, validation, and regulatory/industrial applicability.

To avoid conflating food safety with general digitalization or commercial quality management, this review treats quality, authenticity, and digital-platform studies as food-safety-relevant only when they are linked to exposure reduction, hazard detection, traceability, recall, HACCP verification, fraud-related safety risk, allergen or prohibited-ingredient risk, or official-control decision support. Studies dealing only with sensory quality, yield optimization, consumer preference, sustainability, or generic automation are considered contextual and are not used as evidence of food-safety control maturity.

### 1.1. Scope, Objectives, and Guiding Questions

The aim of this review is to critically examine the control functions in which AI can add value to postharvest food-safety management of animal-source foods, and under what conditions it can move beyond laboratory or pilot-stage demonstration. The narrative synthesis is guided by four questions: (i) at which postharvest control points is the scientific and practical rationale for AI-based solutions strongest; (ii) what type of evidence is required before a system can support a food-safety decision; (iii) what are the main organizational, data-governance, and regulatory barriers to implementation; and (iv) which publication and research priorities would strengthen the maturity of the field. The output of the review is therefore not a formal evidence map or meta-analysis, but an interpretive and practical decision framework for distinguishing proof-of-concept papers from near-implementation studies.

Three editorial and scientific problems motivated this review. First, the 2023–2025 wave of reviews is substantial but fragmented: broad AI–food-safety literature, slaughterhouse computer vision, HACCP monitoring of animal-source food, seafood safety, and traceability/food-fraud technologies are developing along partly separate lines [9,10,11,12,13,14]. Second, these reviews are typically organized around technologies or subfields, whereas veterinary food-safety and official-control readers usually want to understand a decision context—for example, prescreening, support for official control, improved recall precision, or digital verification. Third, the field often conflates high algorithmic performance with implementability, even though validation, governance, and workflow integration form the critical gap between them. Accordingly, the review applies a control-oriented evidence-maturity framework to distinguish proof-of-concept demonstrations from applications that are closer to validated implementation.

### 1.2. Narrative Review Approach and Source-Selection Logic

This narrative review was designed with structured literature mapping rather than as a systematic review, scoping review, or meta-analysis. This format was selected because the relevant literature is methodologically heterogeneous: it includes primary predictive studies, sensor and packaging developments, digital traceability papers, official-control and governance articles, and broad as well as highly focused reviews. The aim was therefore to develop an interpretive synthesis of application maturity, validation gaps, and implementation bottlenecks rather than to estimate a pooled effect size or to exhaustively catalog every AI application in food safety.

The literature was identified through structured searches and iterative citation tracking. Searches were performed in Scopus, Web of Science, PubMed/MEDLINE, and Google Scholar using combined terms from three blocks: (i) artificial intelligence, machine learning, deep learning, computer vision, Internet of Things, sensor, digital traceability, blockchain, or decision support; (ii) meat, milk, dairy, egg, seafood, fishery product, or animal-source food; and (iii) food safety, postharvest control, slaughterhouse inspection, cold chain, HACCP, traceability, authenticity, recall, validation, auditability, or regulatory applicability. Peer-reviewed publications published mainly between 2020 and 2025 were prioritized, while earlier methodological, validation, and official-control papers were retained when they were foundational to the topic.

Titles and abstracts were first screened for relevance to postharvest food-safety control. Potentially relevant full texts were then assessed for at least one of the following elements: animal-source food matrices, a postharvest control point, a human food-safety or zoonosis-control endpoint, a reference method, internal or external validation, workflow integration, auditability, traceability, or regulatory/official-control relevance. Papers were excluded when they focused only on crop production, farm-level production without postharvest control, generic digitalization, education, consumer preference, sustainability, or commercial quality attributes without a clear food-safety or regulatory endpoint. Because this was a narrative review rather than a systematic review, no pooled effect estimate or formal risk-of-bias score was calculated.

The narrative synthesis was organized by control function—slaughterhouse and plant prescreening, cold-chain and condition monitoring, traceability, recall and authenticity, and digital HACCP verification—because these categories reflect decision contexts rather than technology classes. The transparency and internal quality of the review argument were checked against narrative-review quality principles, especially clarity of aims, justification of source selection, appropriate referencing, scientific reasoning, and balanced presentation of limitations [15,16,17].

### 1.3. Contribution Relative to Recent Reviews

This section defines how the present review is positioned relative to recent AI–food-safety reviews and clarifies the analytical filters used in this review [8,9,10,11,12,18,19]. These studies are important, but because their analytical units differ, none of them brings postharvest control settings of animal-source food, evidence thresholds, and regulatory/industrial implementability into a single framework.

The specific objective of the present review is to integrate postharvest control functions, evidence thresholds, validation requirements, auditability, and regulatory/industrial implementability into one practical framework for animal-source food safety. First, it organizes the literature by control function rather than by technology class, which is closer to real decision contexts in postharvest food-safety control. Second, it elevates to the main analysis only those applications that are linked to animal-source food matrices and human postharvest food-safety endpoints. Third, it evaluates external validation, reference methods, auditability, workflow integration, and regulatory readiness in a consistent manner across use cases. Fourth, as a narrative synthesis, the endpoint of the review is not simple trend description but the formulation of practical minimum requirements and decision filters for evaluating AI-based animal-source food control systems. Table 1 briefly summarizes how the present review differs from the closest recent reviews.

## 2. Food Safety Logic of Postharvest Control in Animal-Source Food Chains

### 2.1. Major Hazards and Critical Control Points

In animal-source food chains, risks do not concentrate at a single point; they accumulate across several critical transitions from production to consumption. Slaughterhouse operations, cutting, heat treatment, pasteurization, packaging, post-process contamination, storage, and chilled logistics are all nodes where microbiological hazards can intensify rapidly, while raw material origin, lot mixing, and missing data make source identification more difficult [3,4,20,21]. Both the risk-based meat safety assurance literature and broader risk-based inspection literature indicate that weak points in the control system lie not only in the hazards themselves, but also in decision delay, fragmented documentation, and poorly targeted use of inspection resources. Table 2 summarizes the animal-source food commodities and control points where digital or AI-based approaches appear most relevant.

**Figure 1:**
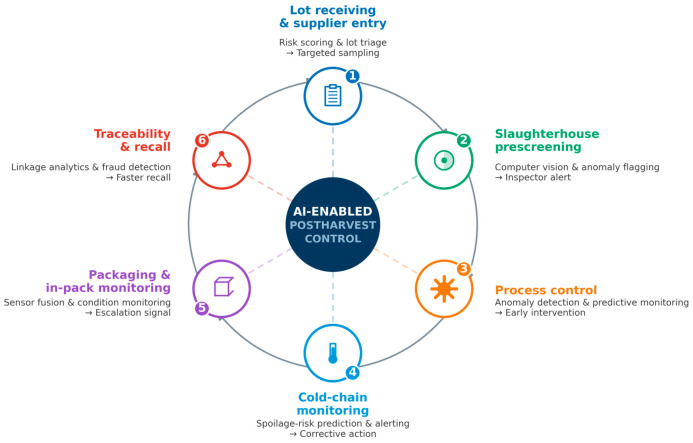
Workflow view of AI-enabled postharvest control points across animal-source food chains.

The same synthesis can also be condensed into a dedicated visual (Figure 1).

### 2.2. Why Is Conventional Control Insufficient?

A major strength of conventional food-safety management is its well-established sampling, analytical, and documentation routine, but its weakness lies in fragmented data structures, delayed feedback, and cumbersome integration. In many plants, critical data points still reside in separate laboratory, logistics, quality assurance, and audit files, which slows hazard analysis and targeted intervention [5,9,28]. This becomes especially problematic when rapid decisions are needed, for example after a cold-chain breach, a positive environmental sample, a recall, or lot tracing across multiple plants.

The promise of digital systems is not to replace human control, but to increase monitoring density, reduce response time, and connect data in a time-stamped, auditable manner. From a control-implementation perspective, however, it is not enough for a system to be merely continuous or automated; it must also be shown that the additional data improve risk detection, decision quality, traceability, or recovery speed.

## 3. Added Value of AI by Control Function

For the present narrative review, the most useful organizing principle is control function rather than technology class. Along the animal-source food chain, four functions currently show the strongest evidence base: early detection, prediction, traceability, and verification.

Together, these four control functions cover several layers of postharvest food-safety decision-making with different risk profiles. Early detection typically supports prescreening or triage-type decisions; cold-chain monitoring supports time-sensitive alerts and targeted intervention; traceability supports lot identification and recall precision; and digital HACCP supports documented verification and closure of corrective actions. Distinguishing these four functions is important because the same technology—such as computer vision or sensor data analytics—may be considered editorially strong at very different thresholds of proof depending on whether it supports a prescreening alert or a quasi-decision-replacing control function [6,8,29].

### 3.1. Early Detection at Slaughterhouses and Processing Plants

Computer vision applications in slaughterhouses and meat processing plants are among the most tangible AI-use cases. Image-based analysis of meat surfaces, carcasses, and processing operations can support rapid prescreening of lesions, contamination, color deviations, faulty separation, or other visual anomalies, and may also help standardize post mortem inspection [29]. These systems are most promising when linked to a clearly bounded decision point—for example by generating a prescreening alert, triggering re-sampling, or directing the attention of a human inspector.

At the same time, real-world performance in slaughterhouse and plant environments is strongly influenced by lighting, camera placement, type of contamination, processing speed, and species-specific variability. It follows that laboratory accuracy or single-plant validation alone is not sufficient; external validation, stability across sites, and the operational impact of false alerts are critical [8,11].

At the study level, the strongest papers in this area do not merely report image-classification accuracy; they link the imaging endpoint to an actual hygiene or quality decision. In such cases, model performance matters because it can accelerate lot segregation, targeted sampling, the initiation of rewash or cleaning interventions, or the prioritization of human inspection. Weaker evidence typically relies on single-plant datasets collected under tightly controlled lighting and narrow label sets, where real transferability cannot be seen. The strongest editorial position is approached by studies that evaluate the algorithm across multiple shifts, days, or plants and translate error classes into the language of the real control process. Slaughterhouse computer vision is therefore one of the most mature AI application areas in animal-source food safety today, but mainly for prescreening and verification support rather than fully autonomous decision use [12,22].

### 3.2. Prediction and Condition Monitoring in the Cold Chain

The second major application group concerns prediction of pathogen or spoilage risk and real-time detection of temperature and logistics anomalies. IoT-based sensors, smart labels, intelligent packaging solutions, and data-integration platforms make it possible to continuously monitor cold-chain interruptions, storage-time exposure, or deviations in processing parameters [5,26,30,31,32,33,34]. ML models can link these data to microbiological, spoilage, or loss-risk outcomes, which is particularly relevant for dairy, fish, and ready-to-eat meat products. The smart seafood-safety literature shows clearly that the combination of sensorics, image processing, and predictive analytics is strongest where the signal leads directly to an operational alert or shelf-life decision [13].

In this area, practical benefits are already partly visible today: faster alerts, more targeted selection, shorter response times, and retrospectively interpretable data streams can all create direct operational value [18,32]. Even here, however, the evidence is strong only when model performance is compared with real plant deviations rather than purely laboratory simulations, and when it is documented that the alert actually prevented the release of a defective lot, better targeted sampling, or improved recall precision. Studies that connect sensor data to multivariable shelf-life or quality outcomes and then implement these on a real-time platform are particularly valuable [27,35].

Within the condition-monitoring literature, there is a sharp quality difference between systems that merely explain spoilage retrospectively and those that provide a genuinely usable, pre-defined alert signal. The strongest papers build decision-support models from multiple sources—time–temperature curves, humidity, packaging or biosensor signals, and sometimes logistics event data—and make clear what intervention the alert is intended to trigger. Weaker publications, by contrast, often estimate quality change retrospectively and mainly for explanatory purposes, without showing how the model connects to actual sampling, hold/release, or shelf-life decisions. From a postharvest control perspective, the key question is therefore whether the system can reduce response time and improve risk triage, not merely whether it detects a statistical relationship between sensor signals and spoilage [5,26,27,30,32,34,36].

### 3.3. Traceability, Recall, and Authenticity

The third control function is rapid lot identification and evidence-based recall support. Integrated digital traceability systems, and in some cases blockchain-based architectures, make it possible to connect data from multiple points in the chain, which can reduce source-identification time and improve incident response [14,25,37,38,39]. In the animal-source food sector, this is particularly important for products in which lot mixing, multi-supplier inputs, or cross-border movement complicate the determination of contamination origin.

Authenticity and adulteration applications are most relevant to veterinary food safety and official-control practice when quality or origin protection is directly linked to safety, labeling compliance, zoonosis-control relevance, or fraud prevention. Good examples include sensor-plus-AI solutions for milk adulteration and machine-learning systems for meat traceability [22,24]. Here too, however, the value of publications increases when the chemical or origin-verification method used as a reference is clearly stated and model performance is validated on heterogeneous samples from real production settings.

In traceability and authenticity systems, not every digital innovation carries equal weight from a food-safety perspective. A blockchain- or platform-based solution is not in itself a food-safety-control advance if it does not shorten the exposure window, improve recall precision, or support root-cause analysis. The strongest applications are those that organize lot, supplier, laboratory, and logistics data into a common chain of evidence so that the causal reconstruction of an irregularity or contamination event becomes faster. For lot-level traceability and transport visibility, IoT- and blockchain-based systems mainly support food safety when they shorten supplier, logistics, and lot reconstruction during incidents [25,37,39]. For meat traceability and authenticity, machine-learning and sensor-based methods are stronger when they are benchmarked against a defined laboratory, compositional, or origin-verification reference [22,24,40]. Food-fraud applications should be interpreted as food-safety-relevant only where the fraud mechanism creates exposure, allergen, compliance, prohibited-ingredient, or recall-management risk [39,40,41].

### 3.4. Digital HACCP and Decision Support for Verification

The fourth area, and one that is particularly important for veterinary food-safety control, is the digitalization of HACCP systems and decision support for verification. Digital CCP monitoring, automated logging, trend analytics, and risk-based alert logic can in principle strengthen documentation, traceability, and auditability [12,42]. Real benefit arises, however, only when the system is not added as a parallel administrative burden, but genuinely reduces documentation loss, improves early recognition of trends, and supports preventive action.

The literature shows that digital HACCP systems are far from homogeneous. Many solutions are limited to data visualization or checklist digitization, whereas others contain genuine predictive or rule-following components [10,43]. Future publications therefore need to distinguish clearly between mere data digitization and actual AI-based decision support.

For digital HACCP systems, the strongest evidence does not come from dashboard aesthetics but from closure of the control loop. In a truly mature system, it is clear which signal triggers an alert, who approves the intervention, how the correction is documented, how post hoc verification occurs, and how the audit trail is preserved. Yet many papers still present primarily checklist digitization, data visualization, or general digitalization concepts, while decision authority and error-handling rules at critical points remain insufficiently documented. From an editorial perspective, the strongest system is therefore one that handles CCP monitoring, non-conformity management, and verification evidence within a single traceable architecture, and does not blur the distinction between decision support and decision replacement [29,42].

### 3.5. What Should Currently Count as Truly Strong Evidence?

Because this is a narrative review, the evidence levels below should be interpreted as practical evidence-maturity categories rather than as a formal grading system or risk-of-bias assessment. Based on the present literature, the evidence maturity of animal-source food postharvest AI systems can be arranged into five clearly distinguishable levels. At the lowest level are laboratory or highly controlled proof-of-concept studies, where the model performs only under favorable conditions on a narrow dataset. These are followed by single-site retrospective studies, which provide a more realistic picture but still fail to capture seasonal, inter-plant, and operator variability. The third level consists of prospective pilots, where the system runs on an actual operational data stream, even if only over a limited period. The fourth level is multi-site or multi-time-point evaluation, which begins to provide genuine evidence of transferability. The highest—and still rare—level is auditable decision support: cases in which the model’s role, error modes, human oversight, and corrective effects are documented as part of the control system itself [8,10,12].

To make this classification operational, the levels were applied using the following decision rules. Level 1 was assigned when the model was tested only under laboratory, simulated, or highly curated conditions and no real operational data stream was used. Level 2 was assigned when retrospective data from one site, one plant, one device, or one narrowly defined dataset were analyzed, even if internal cross-validation was reported. Level 3 was assigned when the system was evaluated prospectively on an operational data stream, with pre-defined alert logic or decision thresholds, but only for a limited site, period, or matrix. Level 4 was assigned when performance was tested across multiple sites, seasons, years, devices, product matrices, or clearly separated temporal datasets. Level 5 was assigned only when the model was embedded in an auditable decision-support workflow with documented human oversight, error handling, corrective actions, version control, and fallback procedures. When a study met criteria from more than one level, it was assigned to the lower level unless the higher-level validation and workflow conditions were explicitly demonstrated.

On this scale, the strongest current positions are occupied by slaughterhouse visual prescreening, cold-chain anomaly surveillance, and lot-level traceability architectures, because the decision point, intervention logic, and use context are relatively well defined in these areas. The evidence is weaker where AI is assumed to act directly as a product release tool, an automated compliance classifier, or a substitute for official decisions. This is not because such systems are impossible in principle, but because the published literature has not yet shown sufficiently robust multi-site validation, error tracking, and governance transparency. That distinction is an important part of the articles’ novelty: the review ranks not the general potential of AI, but the levels of application maturity that can currently be defended [11,18,19].

## 4. Validation Requirements and Levels of Evidence

The greatest weakness of the field at present is not the lack of technological ideas, but the heterogeneity of validation. A substantial share of papers report excellent accuracy metrics, yet do not describe in sufficient detail the sampling frame, reference method, class prevalence, data partitioning strategy, or external-validation conditions [8,19]. In food-safety control, however, the statement that a model “performed well” is not enough; the consequences of the decisions also need to be understood.

### 4.1. Data Representativeness, Labeling, and Reference Method

The first minimum requirement is a transparent description of the sample set and reference method. For an image-based system, it must be clear from which species, at which processing stage, under what hygiene status, and under which environmental conditions the images were obtained. For a microbiological prediction model, it is equally critical how the outcome variable was defined, whether by a gold standard or at least a reference method, and to what extent the data represent real operational variability [12,44,45]. Weak labeling or a non-comparable reference base can create a false sense of security.

### 4.2. Internal and External Validation

The second key issue is external validation. Train/test separation, cross-validation, and avoidance of data leakage are important, but they do not by themselves prove that the system remains stable in another plant, another year, another shift, or another product category. For food-safety control, greater interpretive weight therefore lies with studies that assess performance across multiple sites, multiple lots, multiple matrices, or at least temporally separated sample sets [8,29].

External validation was therefore interpreted as a gradient rather than as a binary label. Temporally separated testing within the same plant evaluates time extrapolation. Testing on another device, camera, operator, or production line evaluates technical or procedural robustness. Testing in another plant evaluates site extrapolation. Testing across regions, countries, product matrices, or production systems evaluates broader geographic and matrix extrapolation. These forms of validation should not be treated as equivalent: temporal validation is useful but weaker than independent multi-site or multi-region validation for judging regulatory readiness [8,29,45].

Appropriate selection of metrics is equally important. Accuracy alone is often misleading; in food-safety applications, sensitivity, specificity, positive predictive value, negative predictive value, calibration, and the operational cost of false alerts are frequently more informative. For a cold-chain alert system, for example, too many false positives can lead to alert fatigue, whereas false negatives may carry direct public health risk.

Calibration should be treated as a validation requirement distinct from discrimination. This is particularly important in low-prevalence food-safety scenarios, where a model may show acceptable AUC, sensitivity, or specificity but still produce poorly calibrated risk estimates. Poor calibration can lead to inappropriate lot holds, false reassurance, unnecessary sampling, or alert fatigue. Studies should therefore report, where applicable, calibration plots, observed-to-expected event ratios, Brier scores, or other calibration diagnostics, and should describe whether recalibration is needed when the model is transferred to a new plant, season, device, or product matrix. Positive and negative predictive values should also be interpreted in relation to the expected prevalence of the target hazard or deviation [6,44,45].

### 4.3. Workflow Integration, Auditability, and Technology Readiness

The third level concerns proof of real-world operation. A system may be technically accurate and yet unsuitable for inter-plant use if it cannot achieve the required throughput, cannot be integrated into existing IT infrastructure, does not document the model version, or leaves unclear who may override the alert and how. In regulatory or audit settings, verifiability, logging, version control, and human review are therefore not secondary but primary requirements [9,46,47]. Table 3 summarizes these minimum requirements at the decision and documentation level.

**Figure 2:**
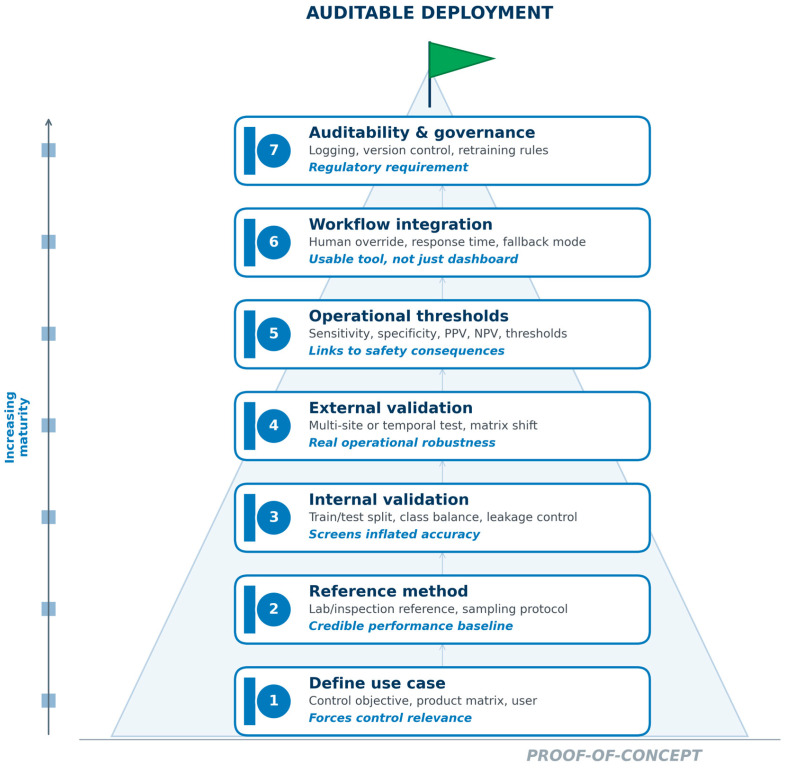
Validation pathway from proof-of-concept to auditable plant deployment.

Figure 2 summarizes the validation pathway used in this review. It should be read as a staged decision pathway rather than as a simple technology-readiness ladder: a system should not progress toward workflow deployment unless the use case, reference method, internal validation, operational threshold, and at least temporal or site-level external validation are documented. Exit criteria between stages include inadequate reference labeling, data leakage, unstable performance across time, site, device, or matrix, unacceptable false-alert burden, missing fallback procedures, unclear human override, or absence of audit logging.

### 4.4. What Makes an AI Study Editorially Strong?

From a veterinary food-safety and official-control perspective, an AI study becomes scientifically strong when it documents the full evidentiary chain of the control decision rather than merely reporting performance metrics. At a minimum, this requires a clear description of the target population and product matrix, identification of the reference method or expert gold standard, the logic of data partitioning and external validation, justification of operational thresholds, and an explanation of which operational step is triggered by the alert. Editors are interested not only in how accurate a model is, but also in where it fails, in whom it fails, with what consequence it fails, and whether a human or organizational control exists that can detect and manage the error. This is especially important when the system targets rare but high-risk events, where accuracy alone may be misleading [8,12,19].

Documentation of failure modes and operational limits is equally essential. Authors should report how performance changes by species, product category, plant, environmental condition, sensor type, or lighting condition, and what happens in cases of network failure, missing data, or model retraining. Truly strong studies do not conceal limitations; they show how the system fails safely, how control reverts to manual procedures, and how auditability is preserved after version changes. This is what turns a study from a technological demonstration into food-safety control science [10,11,29].

## 5. Implementation Constraints and Regulatory Readiness

### 5.1. Data Governance, Interoperability, and Cybersecurity

One of the greatest barriers to implementing digital control systems is that operational, laboratory, logistics, and regulatory data exist in different formats, under different ownership logics, and often with limited shareability. Lack of interoperability constrains risk-based decision support, while cybersecurity vulnerabilities directly threaten system reliability and user trust [48,49,50]. In food-safety control this is not a theoretical issue: a corrupted or attacked data stream may lead to incorrect lot holds, missed alerts, or misleading audit trails.

### 5.2. Human Oversight and Accountability

In postharvest control, AI systems are most legitimately viewed not as autonomous decision makers, but as decision-support or prescreening components operating under human oversight. This is especially true for slaughterhouse inspection, recall decisions, and compliance procedures, where erroneous decisions have major legal, economic, and public health consequences. Algorithmic transparency is therefore not merely an ethical issue but an operational one: the user must understand from which data, and according to which rules or patterns, the alert was generated, and how any override can be documented [46,47].

### 5.3. SMEs, Deployment Constraints, and Market Context

Implementation barriers are not distributed evenly across actors. Large companies are better able to finance sensor networks, system integration, and data-analytics capacity, whereas for SMEs the cost of investment, maintenance, training, and vendor dependence represents a much greater risk. The literature also suggests that, instead of highly complex general-purpose platforms, targeted implementation for a narrow, well-defined problem—for example, automated monitoring of a single critical cold-chain point or a single high-risk inspection location—often offers a better return pathway [10,28,51].

For SMEs, a more realistic implementation pathway is phased and modular adoption rather than full digital transformation at once. Practical mitigation strategies include starting with one high-risk and well-defined control point, using lightweight or open-source analytics where appropriate, relying on validated cloud-based services under clear data-governance contracts, sharing reference datasets or models through industry associations or cooperatives, requiring vendor-neutral data formats, and combining digital alerts with existing HACCP documentation rather than creating a parallel administrative system. Such approaches can reduce entry costs, dependence on a single vendor, and training burden while still improving monitoring density and auditability [10,28,51].

### 5.4. Which Use Cases Are Closest to Regulatory Readiness?

Based on currently published evidence, the use cases closest to regulatory readiness are those that support control faster and more consistently while retaining human approval. These include prescreening of visual or sensor-based anomalies, prioritization of targeted sampling, automatic signaling of cold-chain events, narrowing the set of lots affected during recalls, and integrating verification documents and data streams. In these cases, AI does not act as an autonomous regulatory or operational decision maker, but improves response time, documentation, and consistency. This role is currently much more defensible than fully autonomous classification or release, because decision accountability, human oversight, and the audit trail remain intact [8,29,52].

The meat-inspection and remote-inspection literature is a particularly important benchmark for judging regulatory readiness, because it deals not with generic digital optimism, but with the conditions under which control accountability and the documentation chain can be preserved. The shared message of internal-audit-based official-control development, the risk-based meat safety assurance concept, and remote-inspection studies from Sweden and Finland is that a digital tool can be regarded as regulation-adjacent only if on-site verification, human responsibility, and a legally defensible audit trail are not weakened but strengthened [20,52,53,54,55].

The least defensible use cases, by contrast, are those in which AI would directly trigger product release, condemnation, compliance classification, or shelf-life extension decisions without clearly documented continuous comparison with a reference method, rules for model updates, and a defined fallback mode. High predictive performance is therefore not enough for regulatory applicability; version control, retraining rules, cybersecurity protection, interoperable data exchange, and the ability of an inspector or quality-management actor to reconstruct after the fact which data and which logic produced the alert are also required. Within this framework, generative AI should be considered a secondary documentation and knowledge-management layer rather than a primary control architecture. Potentially useful applications include drafting HACCP records from verified source data, summarizing non-conformity reports, assisting with corrective-action documentation, querying standard operating procedures, translating audit findings, or preparing structured recall documentation. However, generative AI outputs cannot substitute for primary measurements, reference methods, inspector judgment, or legally accountable release and compliance decisions. Any use of generative AI should require human verification against source records, traceable citations or document links, logging of the tool and model version, protection of confidential business data, and safeguards against fabricated or unsupported statements [39,47,48,50,54,55].

### 5.5. Long-Term Maintenance, Drift Detection, and Model Updates

Post-deployment performance should be treated as part of the food-safety control system. AI-supported controls may drift when product mix, suppliers, season, equipment, lighting, cleaning regimes, operators, sampling practices, or prevalence change. Deployment studies should therefore specify input-drift indicators, periodic comparison with reference methods, performance-review intervals, and pre-defined triggers for recalibration, retraining, suspension, or withdrawal of the model [8,10,45,47].

Every model update should be auditable. The active model version, training-data window, validation report, approval date, responsible person, and fallback procedure should be logged. Updated models should be run in parallel with the previous version or with manual control until their performance is verified. Without lifecycle documentation, an AI tool may improve initial detection but weaken long-term regulatory traceability [46,48,50].

## 6. Research and Publication Priorities

Based on the current literature, increasing the maturity of the field will require not primarily more algorithms, but better study designs. First, the number of multi-site, prospective, and ideally workflow-embedded validations must increase. The central question is not whether a model can classify a given dataset, but whether it reduces detection delay, improves sampling targeting, lowers the risk of unnecessary recall, or strengthens the reliability of compliance documentation.

Second, standardized reporting minimums are needed. In animal-source food-safety applications, published performance is difficult to compare unless the product matrix, sampling frame, reference method, type of external validation, decision thresholds, and the consequences of false positives and false negatives are described. The field would benefit from shared benchmark datasets, open annotation rules, and minimum reporting checklists that move papers closer to regulatory and industrial usability. For predictive and decision-support AI systems, transparent reporting frameworks such as TRIPOD+AI offer a useful analogy, even though they originated in biomedicine [12,45].

Finally, regulatory applicability should not be treated as an afterthought. Model governance, retraining rules, version control, audit trail, and human overrideability need to be considered at the study-design stage. The literature on official control and risk-based inspection suggests that implementability must be addressed in the study plan, not only in the final paragraph of the discussion [20,21,52]. Such work is more likely to meet the expectations of veterinary food safety and zoonosis-control readers because it links technological novelty to control practice, auditability, and implementation relevance.

### 6.1. Limitations of the Present Narrative Review

This design was chosen because the relevant evidence base is methodologically heterogeneous and includes predictive modeling studies, sensor and platform papers, implementation studies, governance literature, official-control literature, and prior reviews.

As a result, the synthesis is interpretive and control-function oriented. The main limitations are potential selection bias in the literature discussed, underrepresentation of non-English or unpublished implementation experience, and possible publication bias toward positive AI applications. To reduce these limitations, the review makes its source-selection logic explicit, focuses on peer-reviewed literature mainly from 2020 to 2025, and uses validation, auditability, workflow integration, zoonosis-control relevance, and regulatory applicability as consistent interpretive filters across the literature discussed.

### 6.2. Short- and Medium-Term Research Agenda

The most important task of the next research cycle is not to introduce more isolated algorithms, but to raise evidence thresholds step by step. In the short term, the field needs prospective, preregistered, or at least transparently documented implementation studies in which the control objective, reference method, operational threshold, and corrective decision are specified in advance. In the medium term, the greatest added value will come from multi-site evaluations, harmonized reporting minimums, governance rules, and outcome measures linked to real workflows. Table 4 condenses this priority logic across four leading control functions.

From a publication-strategy perspective, this means that the strongest future studies are likely to be those that present not the largest number of models, but the clearest chain of proof. Studies that test the same application across multiple plants or regions, characterize failure modes, and compare AI-supported control with usual practice in terms of response time, documentability, and risk-reduction benefit will be particularly valuable. Such an agenda is not only scientifically stronger, but also better aligned with the Veterinary Sciences readership because it ties technological novelty directly to veterinary food safety, zoonosis-control relevance, official-control practice, and practical implementation value [8,10,18].

**Figure 3:**
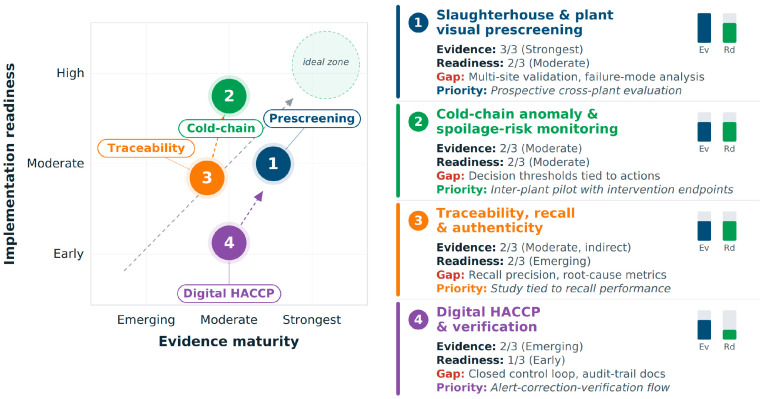
Qualitative evidence-to-implementation roadmap across the main postharvest control functions for animal-source foods.

To provide a more structured study- and technology-level overview, Supplementary Table S1 summarizes the main reviewed application groups, representative references, dominant technologies, validation approaches, implementation limitations, and qualitative evidence-maturity and implementation-readiness scores. Figure 3 was constructed from the qualitative evidence-maturity and implementation-readiness categories applied in Section 3, Section 4, Section 5 and Section 6 and Supplementary Table S1, not from pooled quantitative meta-analysis. For each control function, evidence maturity was scored as 1 = proof-of-concept or highly controlled evidence only, 2 = moderate evidence with prospective, operational, or temporal-validation elements, and 3 = stronger evidence with external, multi-site, or implementation-relevant validation. Implementation readiness was scored as 1 = early-stage applicability, 2 = decision-support readiness under human oversight, and 3 = implementation-proximate use with documented workflow, fallback, and audit requirements. Scores were assigned conservatively on the basis of the cited core studies and were lowered when reference methods, calibration, workflow testing, or governance were insufficiently reported.

## 7. Conclusions

AI offers real but strongly conditional added value in postharvest food-safety control of animal-source foods. The most promising applications appear in well-defined, data-intensive, and time-sensitive control settings: slaughterhouse and processing-plant prescreening, cold-chain and condition monitoring, authentication, traceability, and digital HACCP verification. However, the current literature still too often remains at the proof-of-concept level and pays insufficient attention to how a system performs in another plant, another year, under real workload, or in an audit environment.

The central message is therefore that the success of AI systems will be determined not by laboratory accuracy, but by reliable validation, robust data governance, auditability, interoperability, and safe integration under human oversight. When these conditions are met, AI is not an alternative to conventional food-safety control, but a targeted, risk-based reinforcement of it. The novelty of this narrative review lies accordingly not in presenting another technology catalog, but in making visible where the boundary currently lies between proof-of-concept evidence and implementation-proximate, auditable AI support that is relevant to veterinary food safety, zoonosis control, One Health-oriented risk management, and official-control practice.

## Figures and Tables

**Table 1:** Positioning of the present review relative to closely related reviews published in 2023–2025.

Primary Focus	What Remains Missing from the Perspective of the Present Question?	Added Value of the Present Review
Broad role of AI in food safety [9]	Not specific to postharvest animal-source foods; limited explicit minimum validation requirements	Reorganizes the field by postharvest control functions in animal-source foods and evidence thresholds
Computer vision in meat inspection [11]	Focuses on a single method class; does not compare cold-chain, traceability, and HACCP use cases	Compares multiple control functions within a shared validation and implementation framework
ML in food safety and HACCP monitoring for animal-source foods [12]	Very close thematic review, but with limited explicit filtering for regulatory readiness, audit trail, and workflow integration	Adds an editor-facing evidence framework and minimum validation requirements
Enablers and constraints of FSMS digitalization [10]	System-level perspective rather than application- and matrix-level validation audit	Translates system-level lessons into postharvest publication and implementation criteria for animal-source food
Broad narrative on AI in food-safety/quality management [18,19]	Blends safety, quality, and sustainability endpoints; lacks a veterinary food-safety and official-control decision threshold	Applies simultaneous filters for human food safety, postharvest relevance, and implementability

**Table 2:** Animal-source foods: major hazards, critical control points, and digital/AI-based control opportunities.

Product Group	Main Biological Hazards (ex.)	Main Chemical/Other Hazards (ex.)	Key Control Points (ex.)	Digital/AI-Based Controls (ex.)
Poultry meat [4,11,12]	*Salmonella* spp.; *Campylobacter* spp.	Antimicrobial residues	Farm–transport–slaughter (evisceration)–chilling	ML-based risk prediction from farm and slaughterhouse data; computer vision contamination detection; IoT-based cold-chain monitoring
Pork [11,20,21,22]	*Salmonella* spp.; *Yersinia enterocolitica*; *Trichinella* spp.	Veterinary drug residues	Herd health–slaughter–mincing–heat treatment	WGS/ML-based source attribution; vision-based lesion/deviation detection; digital lot traceability
Beef [11,20,21,22]	STEC; *Salmonella* spp.; *Listeria* spp. (RTE products)	Residues; physical foreign bodies	Hide removal–surface contamination control–mincing	Vision-based/hyperspectral contamination and foreign-body detection; predictive hygiene risk scoring
Milk and dairy products [10,23,24]	*Listeria monocytogenes* (post-process contamination); *Salmonella* spp. (raw milk)	Aflatoxin M1; antibiotic residues	Milking–tank cooling–pasteurization–post-process contamination control	IoT-based tank-temperature and CIP monitoring; ML-based anomaly prediction; sensor+ML adulteration screening
Eggs and egg products [12,19,25]	*Salmonella* spp.	Residues	Production site–grading–washing–storage	Vision-based crack and contamination detection; IoT-based warehouse temperature and logistics monitoring
Fish and seafood [13,26,27]	*Vibrio* spp.; *Listeria* spp.; norovirus (shellfish); *Anisakis* spp.	Histamine; heavy metals	Catch–cold chain–processing–packaging	Smart labels/sensors for freshness tracking; ML-based spoilage and histamine-risk prediction; vision-based defect/parasite signaling

**Table 3:** Minimum validation and implementation requirements for evaluating AI-based animal-source food-safety systems.

Dimension	Elements That Should Be Documented at Minimum	Why Is This Critical?	Typical Weakness in the Current Literature
Sample and matrix description [11,12]	Product category, processing stage, geographic/plant origin, time period, sample size	Representativeness and generalizability cannot be judged without it	Single-plant, curated, or overly homogeneous datasets
Reference method [12,44,45]	Gold standard or comparator laboratory/inspection method; sampling protocol	Model performance can only be interpreted relative to the quality of the reference	Vague labeling; inconsistent reference definition
Internal validation [12,44,45]	Train/validation/test logic, class balance, data-leakage control, repeatability	Prevents overfitting and inflated performance estimates	Accuracy reported without detailed validation description
External validation [11,29,45]	Validation type should be specified: temporal, device/operator, production-line, plant/site, geographic, or product-matrix validation	Different forms of extrapolation carry different evidentiary strength; another year is not equivalent to another plant or region	External validation reported generically without specifying the validation distance or intensity
Operational metrics and calibration [6,45]	Sensitivity, specificity, PPV, NPV, AUC where relevant, calibration plot or observed/expected ratio, Brier score, false-alert rate, decision threshold, and expected prevalence	Reflects both discrimination and the reliability of predicted risk estimates, especially in low-prevalence safety events	Only accuracy or AUC reported; calibration omitted; thresholds not linked to action
Workflow integration [9,10]	Throughput, response time, operator burden, data transfer, fault tolerance	Laboratory performance alone does not guarantee plant usability	No data on real operation or operator burden
Auditability and governance [10,46,47]	Logging, version control, traceability, retraining rules, human override	Primary requirement in regulatory and quality-assurance settings	Black-box character; missing governance description

**Table 4:** Evidence maturity and near-term publication priorities across the main postharvest control functions of animal-source food.

Control Function	Dominant Data Source	What Counts as Strong Evidence Today?	Most Common Weakness	Near-Term Publication Priority
Slaughterhouse and plant visual prescreening [11,29]	Image/video data, process context, expert labeling	Evaluation across multiple shifts or sites; error classes linked to the control process; documented human override	Single-plant datasets; weak transferability; missing laboratory or expert reference	External validation and characterization of real failure modes
Cold-chain anomaly and spoilage-risk monitoring [5,13,27]	Time–temperature data, humidity, IoT events, packaging/biosensor signals	Pre-defined alert threshold; documented intervention; prospective plant pilot	Retrospective models; predictions not linked to action; missing response-time data	Prospective inter-plant studies tied to decisions
Traceability, recall, and authenticity [14,25,37,38,39]	Integrated handling of lot, supplier, laboratory, and logistics data	Demonstrated improvement in recall precision or exposure-window reduction; auditable data links	No explicit safety outcome; only platform description or conceptual architecture	Incorporation of recall metrics and root-cause analysis
Digital HACCP and verification [10,12,42]	CCP logs, sensor data, laboratory results, corrective-action records	Closed control loop: alert, approval, correction, verification, audit trail	Dashboard-level solutions; unclear decision authority and fallback mode	Make the control loop and governance explicit

## Data Availability

The original contributions presented in this study are included in the article. Further inquiries can be directed to the corresponding author.

## Supplementary Materials

The following supporting information can be downloaded at: https://www.mdpi.com/article/10.3390/vetsci13060574/s1, Table S1: Structured overview of reviewed technologies and qualitative evidence/readiness scoring.

## Abbreviations

The following abbreviations are used in this review:

AI	artificial intelligence
AUC	area under the curve
CCP	critical control point
CIP	cleaning-in-place
FSMS	food-safety management system
HACCP	Hazard Analysis and Critical Control Points
IoT	Internet of Things
IT	information technology
ML	machine learning
NPV	negative predictive value
PPV	positive predictive value
RTE	ready-to-eat
SME	small- and medium-sized enterprise
STEC	Shiga toxin-producing Escherichia coli
TRIPOD+AI	Transparent Reporting of a multivariable prediction model for Individual Prognosis Or Diagnosis + Artificial Intelligence
WGS	whole-genome sequencing
