# Supplementary material for: Artificial Intelligence in Postharvest Food Safety Control of Animal-Source Foods: Evidence Thresholds, Validation, and Regulatory Applicability
Source: Vet Sci. 2026 Jun 11;13(6):574. doi: 10.3390/vetsci13060574 (PMC13308038; doi:10.3390/vetsci13060574)
Supplement: Supplementary file 1 [file vetsci-13-00574-s001.zip › vetsci-4342990-supplementary.pdf]

Supplementary Table S1. Structured overview of reviewed technologies and qualitative evidence/readiness scoring

Application group	References	Matrix / context	Main AI or digital approach	Evidence / validation type	Evidence score	Readiness score	Main implementation limitation
Slaughterhouse and plant visual prescreening	[11,29]	Meat inspection; processing plants	Computer vision; image/video analysis	Review and application-level evidence; external validation often limited	3	2	Site transferability, lighting variability, and failure-mode reporting
Meat traceability and carcass classification	[22]	Lamb carcasses; meat traceability	ML-based classification	Case-study ML validation	2	2	Broader breed, site, and geographic validation needed
Cold-chain and logistics monitoring	[5,25,32]	Transport; storage; temperature-sensitive products	IoT sensors; real-time tracking; sensor analytics	Review, platform, and sensor-monitoring evidence	2	2	Alert thresholds not always linked to corrective actions
Smart labels and freshness monitoring	[26,30–36]	Fish, seafood, packaged foods	Smart labels; biosensors; intelligent packaging	Mainly experimental and platform-level evidence	2	1–2	Limited plant-level validation and regulatory linkage
ML shelf-life prediction	[13,27]	Marine fish; seafood chains	ML shelf-life prediction; real-time platform	Species-specific modelling and platform development	2	2	Stronger links needed to hold/release decisions
Dairy process and adulteration monitoring	[23,24]	Milk and dairy products	ML process models; XAI-enabled multi-sensor systems	Review and experimental sensor evidence	2	2	External validation across plants, seasons, and devices needed
Digital traceability and blockchain systems	[14,25,37–39]	Agri-food and livestock-product supply chains	Blockchain; IoT traceability; digital platforms	Review, platform, and case-oriented evidence	2	2	Safety value depends on recall precision and exposure-window reduction
Food fraud and authenticity applications	[24,39–41]	Milk, meat, authentication, fraud prevention	Sensor-based authentication; AI-supported fraud detection	Review and application-level evidence	2	1–2	Safety relevance must be separated from commercial authenticity
Digital HACCP and FSMS digitalization	[10,12,42,43]	HACCP; FSMS; food manufacturing	Digital CCP logs; dashboards; rule-based alerts	Review and system-level evidence	2	1–2	Often lacks closed-loop alert–correction–verification workflow
AI governance and accountability	[47,48]	Food-sector AI governance	Ethics frameworks; AI risk-management concepts	Governance and regulatory literature	2	2	Needs translation into plant-level audit rules

Cybersecurity and data integrity	[49–51]	Food and beverage sector; supply chains	Cybersecurity frameworks; incident reviews	Sector-specific cybersecurity literature	2	2	Often separated from food-safety validation and auditability
Official control and remote inspection	[20,21,53–55]	Meat inspection; official control	Risk-based and remote inspection frameworks	Official-control and inspection literature	3	2	Must preserve legal accountability and defensible audit trails

Note: Evidence maturity score: 1 = proof-of-concept or highly controlled evidence; 2 = limited operational, retrospective, temporal, or single-site evidence; 3 = stronger external, multi-site, or implementation-relevant evidence. Implementation readiness score: 1 = early-stage applicability; 2 = decision-support readiness under human oversight; 3 = implementation-proximate use with documented workflow, fallback, and audit requirements. The 1–3 scores used in this table and in Figure 3 represent aggregated application-level categories and should not be confused with the five study-level evidence-maturity levels described in Section 3.5.
